# Supplementary material for: Hi-LASSO: High-performance python and apache spark packages for feature selection with high-dimensional data
Source: PLoS One. 2022 Dec 1;17(12):e0278570. doi: 10.1371/journal.pone.0278570 (PMC9714948; doi:10.1371/journal.pone.0278570)
Supplement: S1 File — (PDF) [file pone.0278570.s001.pdf]

S1. Simulation study

Table S1. Notations

Notation	Description
n	The number of observations (samples)
p	The number of predictors (features)
q	The number of predictors to be selected in each bootstrap sample
L	The expected value at least how many times a predictor is selected in a bootstrapping.
B	The number of bootstrap samples. To ensure each predictor selected at least L times on average, B can be considered as $B = L \times \frac{p}{q}$.
$\beta = \{\beta_1, \dots, \beta_p\}$	A vector of coefficients.
α	Significance level (e.g., 0.05 or 0.01) in test of significance for feature selection

We generated some simulation data based on a linear regression model, $\boldsymbol{y} = \beta_1\boldsymbol{x_1} + \beta_2\boldsymbol{x_2} + \cdots + \beta_p\boldsymbol{x_p} + \boldsymbol{\epsilon}$, where $\boldsymbol{\epsilon} \sim \mathrm{N}(0, \sigma^2)$ and $\boldsymbol{x_i} \sim N(0,1)$. We considered $\sigma = 3$ at this study. See the notations in Table S1. We considered six simulations' datasets, while varying the numbers of samples, features, and ground truths of the coefficients. The regression coefficients of ground truth ($\boldsymbol{\beta}$) were given in the simulation dataset. The following are the details of the six datasets.

Dataset I consist of 100 variables and 50 samples, where the first 10 coefficients are non-zeros and the remaining 90 coefficients were set to zero. The regression coefficients of ground truth were defined as:

$$\beta = (3, 3, -3, 2, 2, -2, 1.5, 1.5, -1.5, 0, \dots, 0)$$

The pairwise correlations between the first three variables were set to be 0.9, and the same correlation structure was repeatedly set for the next three and four variables. The remaining 90 variables were designed independent from each other. Then, the independent variables $(\mathrm{x}_1, \dots, \mathrm{x}_\mathrm{p})$ were generated from the multivariate normal distribution with zero mean and the covariance matrix of:

$$\begin{bmatrix} \boldsymbol{\Sigma}_{0.9}^{3} & 0 & 0 & 0 \\ 0 & \boldsymbol{\Sigma}_{0.9}^{3} & 0 & 0 \\ 0 & 0 & \boldsymbol{\Sigma}_{0.9}^{4} & 0 \\ 0 & 0 & 0 & \mathbf{I}^{90} \end{bmatrix},$$

where $\boldsymbol{\Sigma}_v^k$ is a $k \times k$ matrix with unit diagonal elements and non-diagonal elements of value v, $\mathbf{I}^k$ is a $k \times k$ identity matrix. Dataset II was generated with double the sample size of Dataset I in the same setting. Dataset III included 1,000 variables and 100 samples, where the first 50 coefficients of non-zero were drawn from $N(0,4)$, and the remaining 950 coefficients were set to zero. We defined a covariance matrix ($\boldsymbol{\Sigma}$) for the first 50 non-zero coefficients on all the datasets, so the variables are highly correlated with various signs. Samples were generated from a multivariate normal distribution with zero mean and the covariance matrix of:

$$\begin{bmatrix} \boldsymbol{\Sigma}_{0.9}^{15} & 0 & 0 & 0 \\ 0 & \boldsymbol{\Sigma}_{0.9}^{15} & \mathbf{J}_{\mathbf{0.3}} & 0 \\ 0 & \mathbf{J}_{\mathbf{0.3}}^{T} & \boldsymbol{\Sigma}_{0.9}^{15} & 0 \\ 0 & 0 & 0 & \mathbf{I}^{950} \end{bmatrix},$$

where $\mathbf{J}_u$ is a matrix with all unit elements of a value u. Dataset IV was generated with double the sample size of Dataset III in the same setting. Dataset V was comprised of 10,000 variables and 200 samples, where the first 50 non-zero coefficients were drawn from $N(0,4)$ and the remaining 9,950 coefficients were set to zero. The setting of Dataset V was identical to Dataset III, but Dataset V included ten times more variables than Dataset III. Dataset VI considered double samples in the same setting of Dataset V. For all the datasets, the signal-to-noise ratios (SNR) corresponding to each dataset are 25.36, 25.68, 4.13, 21.41, 19.05 and 17.4, respectively, where the SNR is defined as $Var\left(\boldsymbol{X}'\boldsymbol{\beta}\right) / Var(\boldsymbol{\epsilon})$.

LASSO, ElasticNet, and Adaptive LASSO were implemented using the Python package, *glmnet*. Precision LASSO and Relaxed LASSO were conducted using their published Python/R packages,

thePrecisionLasso and *relaxo*, respectively. The Random LASSO, Recursive Random LASSO, and Hi-LASSO were implemented in Python.

Table S2. Description of the simulation data

	Number of predictors (p)	Coefficients of ground truth (β)	Number of samples (n)
Dataset I	100	$(3, 3, -3, 2, 2, -2, 1.5, 1.5, -1.5, 0, \dots, 0)$	50
Dataset II	100	$(3, 3, -3, 2, 2, -2, 1.5, 1.5, -1.5, 0, \dots, 0)$	100
Dataset III	1,000	$\beta_1, \dots, \beta_{50} \sim N(0, 4)$, others zero.	100
Dataset IV	1,000	$\beta_1, \dots, \beta_{50} \sim N(0, 4)$, others zero.	200
Dataset V	10,000	$\beta_1, \dots, \beta_{50} \sim N(0, 4)$, others zero.	200
Dataset VI	10,000	$\beta_1, \dots, \beta_{50} \sim N(0, 4)$, others zero.	400
