# Supplementary material for: Hi-LASSO: High-performance python and apache spark packages for feature selection with high-dimensional data
Source: PLoS One. 2022 Dec 1;17(12):e0278570. doi: 10.1371/journal.pone.0278570 (PMC9714948; doi:10.1371/journal.pone.0278570)
Supplement: S2 File — (PDF) [file pone.0278570.s002.pdf]

S2. Performance for efficiency

Hi-LASSO involves high-cost computations caused by a number of bootstrapping for the robust statistical significance test. The computations on the bootstrapping are independent, so the implementation of Hi-LASSO using parallelization can provide efficient solutions for performing Hi-LASSO with high-dimensional data. Also, the implementation of Hi-LASSO using Apache Spark engine provides a scalable solution for large-scale data processing. The parallel version of Hi-LASSO was implemented by using the function ‘concurrent. concurrent.futures.ProcessPoolExecutor’ in the Python library to process parallel multiprocessing to reduce the computational time. The Spark version of Hi-LASSO was implemented using the PySpark, which is a Python library in Apache Spark, to work with huge sets of data.

We compared the execution times of Hi-LASSO with the original implementation with a single process, Hi-LASSO with multiple parallel processing, and Hi-LASSO on Spark with a simulation dataset (Dataset V). We measured the execution time of the two versions of Hi-LASSO on the Intel Xeon Gold 6248R (24 cores x 2) machine and repeated the experiments ten times. The experimental results are shown in Table S3. In the experiments, the implementation of Hi-LASSO with Apache Spark shows the best efficiency (4.83 faster than the single process on 96 processor) to execute Hi-LASSO with a simulation dataset.

Table S3. Execution times of Hi-LASSO with Dataset V.

Number of Processors	Spark (seconds)		Parallel (seconds)
1	2589.51 ± 16.97		4578.14 ± 19.92
12	1062.74 ± 4.66		1788.76 ± 27.6
24	934.14 ± 3.66		1716.39 ± 25.15
48	695.3 ± 3.54		1447.34 ± 43.30
96	536.37 ± 3.9		1219.7 ± 63.43
