# Supplementary material for: Hi-LASSO: High-performance python and apache spark packages for feature selection with high-dimensional data
Source: PLoS One. 2022 Dec 1;17(12):e0278570. doi: 10.1371/journal.pone.0278570 (PMC9714948; doi:10.1371/journal.pone.0278570)
Supplement: S3 File — (PDF) [file pone.0278570.s003.pdf]

S3. Tuning the hyper-parameters in Hi-LASSO

Optimizing hyper-parameters is critical to the performance of feature selection in Hi-LASSO. Hi-LASSO includes three hyper-parameters: (1) the regularization parameter (λ) for (adaptive) LASSO on each bootstrapping; (2) the number of predictors to be randomly selected in each bootstrapping (q_1 and q_2, or simply q when $q = q_1 = q_2$); and (3) the number of bootstrapping samples (B). The optimal λ, which controls overfitting in bootstrapping, was obtained by minimizing prediction errors with *k*-fold cross validation in each bootstrapping. For optimizing the parameters of q and B, we provide empirical insights with experiments, since there is no standard strategy to optimize those hyper-parameters. The smaller q causes more bootstrapping trails, which results in computational cost, whereas the larger q may make coefficient estimation inaccurately, due to the more severe multi-collinearity. B is determined by L (i.e., $B \geq \frac{Lp}{q}$), which indicates at least how many times each variable is selected in the bootstrapping. If B is not large enough, some predictors may not be selected in the bootstrapping, regardless their significance.

We conducted simulation experiments to investigate how the hyper-parameters affect the performance of Hi-LASSO for feature selection. To evaluate the performance of feature selection, we measured F1-scores, varying different combinations of the parameters. We repeated the experiments ten times by randomly generating data, and computed the average of F1-scores. Fig. S1 illustrates F1-scores with various values of *q* and *L*. We considered q = {50, 100, 150, 200, 250, 300, 350, 400, 450, 500} and *L*={10, 50, 100}. Dataset III and Dataset VI showed optimal performance (F1-scores = 0.2803 $\pm$ 0.0892 and 0.8263 $\pm$ 0.0429, respectively), when *q* was equal to the sample size (*q* =100 with Dataset III and *q* = 400 with Dataset VI). Dataset IV showed optimal performance (F1-score = 0.8439 $\pm$ 0.0312) when q = 150, and Dataset V showed optimal performance (F1-score = 0.7126 $\pm$ 0.0279) when q = 300. We empirically found that the optimal values of q were around the size of the sample. The larger *L* generally improved the performance in the experiments but increased the computational cost. However, *L* > 50 does not improve the performance significantly in any settings. We repeated the experiments using the semi-real simulation data, where we set *L*=30 for the sake of simplicity, which approximate normal distribution by the central limit theorem when the distribution is unknown. Fig. S2 shows that the optimal *q* is also around the sample size.

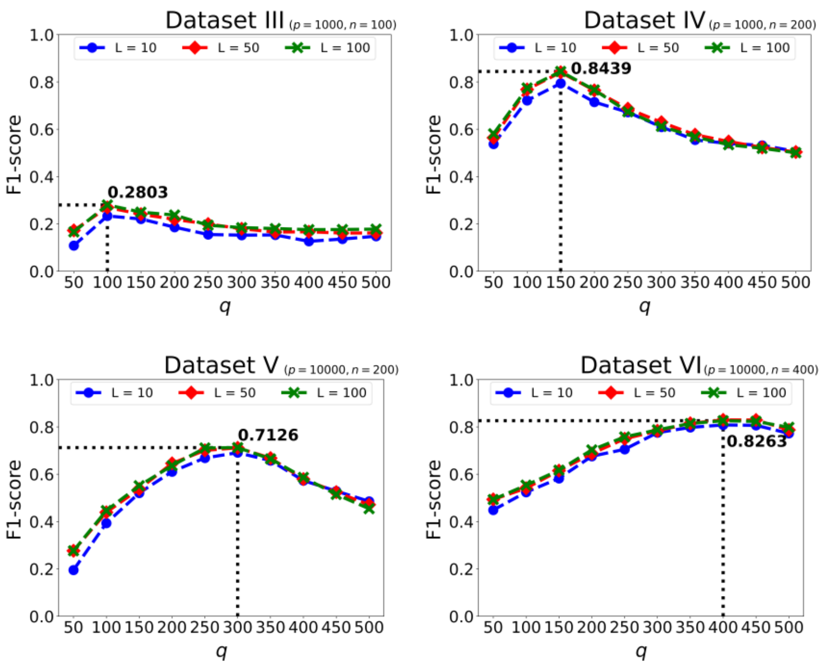

Fig. S1. F1-scores with various values of *q* and *L*.

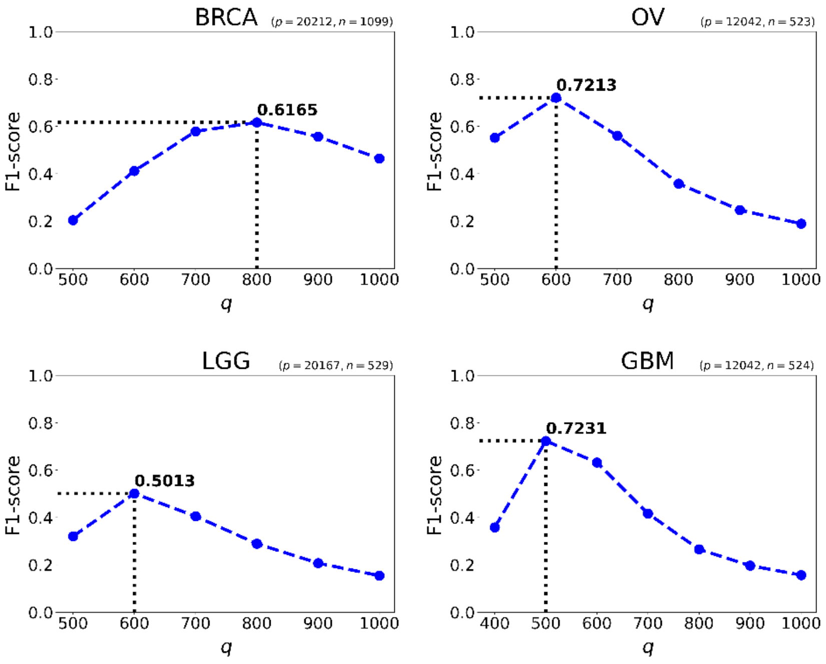

Fig. S2. F1-scores with various values of *q*.
