# Supplementary material for: Hi-LASSO: High-performance python and apache spark packages for feature selection with high-dimensional data
Source: PLoS One. 2022 Dec 1;17(12):e0278570. doi: 10.1371/journal.pone.0278570 (PMC9714948; doi:10.1371/journal.pone.0278570)
Supplement: S4 File — (PDF) [file pone.0278570.s004.pdf]

S4. Robustness analysis using Kuncheva Index (KI)

The robustness of Hi-LASSO's feature selection was evaluated by the Kuncheva Index (KI) since bootstrapping-based methods may provide different result on every execution. The KI computes a stability score in the range of [-1, 1], where zero indicates that two lists are independently drawn, a positive value indicates the feature selection method produces a stable set, and a negative value indicates the method is unstable. The equation of KI is:

$$\mathrm{S_A}(s_i, s_j) = \frac{\left|S_i \cap S_j\right| - \frac{|S_i| * |S_j|}{m}}{\min\left(|S_i|, \left|S_j\right|\right) - \max(0,\ |S_i| + \left|S_j\right| - m)}$$

where S_i and S_i are two given sets, and m is the dimensionality of the feature.
